# Biochemical Characterization of Some Varieties of Apricot Present in the Vesuvius Area, Southern Italy

**DOI:** 10.3389/fnut.2022.854868

**Published:** 2022-03-08

**Authors:** Florinda Fratianni, Rosaria Cozzolino, Antonio d'Acierno, Maria Neve Ombra, Patrizia Spigno, Riccardo Riccardi, Livia Malorni, Matteo Stocchero, Filomena Nazzaro

**Affiliations:** ^1^Institute of Food Science, National Research Council (ISA-CNR), Avellino, Italy; ^2^Cooperativa “ARCA 2010”, Acerra, Italy; ^3^Department of Women's and Children's Health, University of Padova, Padova, Italy

**Keywords:** apricots, polyphenols, ascorbic acid, β-carotene, antioxidants, VOCs, biodiversity

## Abstract

The witnesses of the millenary history of *Campania felix* in southern Italy highlighted that several fruit and vegetables cultivated in such territory could potentially be a treasure trove of important health elements. Our work evaluated the content of β-carotene, ascorbic acid, and total phenolics and the antioxidant activity of ten typical varieties of apricots cultivated in the Vesuvius area in the Campania region. The total polyphenols varied between 10.24 and 34.04 mg/100 g of a fresh sample. The amount of ascorbic acid also varied greatly, ranging from 2.65 to 10.65 mg/100 g of a fresh product. B-Carotene reached values up to 0.522 mg/100 g of the fresh sample. The correlation analysis performed, accounting for these parameters, showed that the antioxidant activity, calculated by 2,2-diphenyl-1-picrylhydrazyl (DPPH assay) and azino-bis (3-ethylbenzothiazoline-6-sulfonic acid) tests, was influenced mainly by the content of total polyphenols, with ρ = −0.762 and ρ = 0.875 when we considered DPPH and ABTS tests, respectively, slightly less by the content of ascorbic acid, and not by β-carotene. The dendrogram clustered eight varieties into two main groups; on the other hand, two varieties (“Vitillo” and “Preveta bella”) seemed hierarchically distant. The gas chromatography–mass spectrometry (GC–MS) analysis of volatile organic compounds (VOCs), herein performed for the first time, demonstrated the influence of the varieties on the VOC profiles, both from a qualitative and semiquantitative perspective, discriminating the varieties in different clusters, each of which was characterized by specific notes. α-Terpinolene was the only terpene identified by GC–MS that appeared to affect the antioxidant activity.

## Introduction

Apricot [*Prunus armeniaca* L.] is a member of the Rosaceae family, and it is cultivated all over the world (1). Apricots contain many secondary metabolites, such as phenolic compounds (2), carotenoids (3–7), in particular β-carotene, and ascorbic acid (8–10). Also present in apricot are seed oils, which are essential for antioxidant activity, and, along with the pectin and mineral elements also contained in these fruits, they promote human health and affect fruit color and taste (11–14). In addition to their functional ingredients, apricots and their derivatives are characterized by distinctive and alluring flavors, to which the presence of volatile compounds also contributes, favouring their consumption worldwide (15, 16). Italy represents the first European country to be a producer of apricots (17); the production is concentrated mainly in Campania, particularly in the Vesuvius area, where we find ~60–80% of the regional production. Herein, apricots are cultivated on ~2,000 hectares, giving rise to ~400,000 quintals of fruit, which are consumed as fresh products, processed in local plants and transformed principally into juices, nectars, jams, etc. The first descriptions of the presence of apricots in Campania are from the Neapolitan scientist Gian Battista Della Porta, who described in his book *Suae Villae Pomarium* dated in 1583 two kinds of apricots, “bericocche” and the better-quality apricots “crisomele,” transformed in the Neapolitan slang to “crisommole,” which is still used today to indicate apricots (18). In the book “Breve ragguaglio dell'Agricoltura e Pastorizia del Regno di Napoli” (A brief overview of the Agriculture and Pastoralism of the Kingdom of Naples)” written in 1845 (19), the apricot tree was identified as the most common tree after the fig tree in the Neapolitan area, particularly the Vesuvius area, “where it grows better than elsewhere, and you can count more kinds of different fruits” (20). In recent centuries, several ecotypes have offered different fruits according to the characteristics of their varieties. Today, over 70 ecotypes of apricots are recognised; they are all grouped under the denomination of “Vesuvius apricot” as they all originate from this area. Vesuvius is an area known for the remarkable fertility of the soils, which, being volcanic, are rich in minerals, particularly potassium, an element known for its influence on the organoleptic quality of the fruit. In this case, potassium contributes to a pleasant and characteristic flavor to apricots. The names of apricot varieties are curious and represent the testimony of a careful selection activity carried out over the centuries by Vesuvius farmers in various fields of cultivation. They include “Boccuccia,” “Pellecchiella,” and “Vitillo,” to name just a few. Recent research has confirmed the high aptitude of Vesuvius apricots for processing into juices and syrups due to the high-sugar content (21). Apricots of the Vesuvius area show characteristics, such as color, texture, and flavor, that make them suitable for use mainly as ingredients in sweets and pastries (22), and they are distributed to regional markets (23). The territory particularly interested in their production includes specific municipalities in the province of Naples, surrounding some sides of the volcanic slopes. Starting from the 1970's, due to the continuous urbanization of the whole Vesuvius area, there was a real downsizing of agricultural activities, which transformed the cultivation of apricots into real tiny orchards often enclosed between buildings. In addition, despite the quality of such apricot varieties, imported apricots are exploited more at the industrial level as fresh products and for the production of derivatives due to their more constant availability (22). However, with the emergence of new markets and new solutions, the focus has recently shifted to the development of new modern plants capable of accelerating cultivation. On the other hand, in recent years, different programs and activities of research have tried to value and safeguard Vesuvius apricots (22–24) by identifying parameters and specific features of these landraces, which can indissolubly bind them to the territory and concurrently enable the identification of the Vesuvius area through the biochemical and nutritional characterisation of such landraces. Thus, due to the wide number of existing varieties present in the Vesuvius area, their characterisation acquires particular importance for both breeders and consumers, who can select and consume specific apricots with a unique flavor and high nutritional quality. Different studies have reported the molecular and qualitative characterisation of apricot landraces of the Campania region and the Vesuvius area (25–28). However, at least, to our knowledge, biochemical analyses of the apricot varieties typical of this territory have not been conducted, and no study has investigated the volatile organic compounds of such varieties.

Therefore, we directed the present study toward the following aims:

To analyse the content of total polyphenols, ascorbic acid, and β-carotene of ten traditional varieties of apricots of the Vesuvius area and determine their antioxidant activity by DPPH radical-scavenging activity and ABTS tests.

Gas chromatography–mass spectrometry (GC–MS) was used to analyse the volatile compounds (VOCs) of the varieties from both qualitative and semiquantitative perspectives and to investigate the VOC dataset through principal component analysis (PCA).

To correlate the antioxidant activity of these varieties with total polyphenols, ascorbic acid, β-carotene, and terpenes identified by GC–MS, we aimed to identify the principal factors affecting the antioxidant properties of the products.

## Materials and Methods

### Plant Material

The analyses were conducted on ten typical varieties of apricots (Prunus armeniaca) obtained from three farms located within the area of Vesuvius National Park. In particular, Egizio Farm located in Somma Vesuviana provided the varieties “Baracca” and “Puscia.” The AGER farm located in Somma Vesuviana offered the varieties “Boccuccia Liscia,” “Caiana,” “Ceccona,” “Pellecchiella,” “Presidente,” “Preveta Bella,” and “Vollese.” Ascione Farm located in Pollena Trocchia supplied the variety “Vitillo.” The two municipalities, Pollena Trocchia and Somma Vesuviana, are <10 km apart and are located on the same side of the volcano. Apricots were immediately taken to the laboratories of the Institute of Food Science and subjected to various analyses. Herein, fruits were pitted, and they were cut into pieces with the whole peel and made into a puree with the help of a home blender in the laboratory. The prepared purees were immediately used for the determination of the contents of total polyphenols, ascorbic acid, and β-carotene.

### Polyphenol Extraction and Quantification

A solution of methanol:acetic acid 99%:1% v/v was added to 10 g of a sample in a 2:1 v/w ratio (28). Samples were perfectly pounded and left for 24 h in the dark at 4°C. Then, they were centrifuged at 4 °C at 11,600 g (Biofuge, Beckman, Cassina de Pecchi, Italy) for 10 min. The removed, filtered, and dried supernatant was then resuspended in 10 ml of 80% ethanol, and ethyl acetate was added. This last step was repeated three times. The organic fraction obtained was then treated with sodium sulphate anhydrous, filtered, and dried again. The residue was resuspended in 1 ml of 50% methanol and stored at −26°C until the analysis. The colorimetric analysis of total phenolics followed the method of Singleton and Rossi (29) with Folin–Ciocalteu phenol as a reagent. The absorbance at λ = 760 nm was determined at room temperature with a Cary UV/Vis spectrophotometer (Varian, Palo Alto, CA, USA). Quantification was based on a standard curve created using gallic acid (y = 0.497 x + 0.0239 R^2^ = 0.9908). The results are expressed as mg of gallic acid equivalents (GAE)/100 g of fresh weight (FW) of the product ± SD.

### Ascorbic Acid Content

Samples were kept in three volumes of 4% metaphosphoric acid in the dark for 1 h at 4°C. After centrifugation at 11,600 x g at 4°C for 10 min (Biofuge, Beckman Italia), the supernatant was collected and filtered (0.45-μm mesh, Millipore, Milano, Italy). The dosage of ascorbic acid was determined by HPLC-UV (Gold System, Beckman Italia, Cascina dè Pecchi, MI, Italy), following the method of Fratianni et al. (28) with a Khromasil KR 100-5 C18 column (mobile phase = sulfuric acid,0.001 M in pure HPLC-grade water; detection wavelength = 245 nm; flow rate: 1. ml min−1). The results were obtained with respect to ascorbic acid used as a standard and are indicated as mg 100 g−1 of a fresh sample.

### β-Carotene Content

Samples were first mixed with 100% ethanol (1:1 w/v), and then petroleum ether (1.5:1 v/v) was added (30). The mixture was vigorously blended and centrifuged (11,600 × g, 15 min; Biofuge, Beckman Italia), and then we recovered the supernatant. The steps were repeated until complete loss of colour was achieved; then, the supernatants were joined together. The quantity of carotenoids was evaluated at λ: 450 nm (Varian) using petroleum ether as a blank and ε = 2,592 as the extinction coefficient. The results are indicated as mg of β-carotene 100 g−1 of a fresh sample.

### Antioxidant Activity

#### Radical-Scavenging Activity

Samples were soaked in methanol (1:3 w/v, plus acetic acid, 1%) and maintained overnight at 4°C (28). The supernatants were recovered (11,600 g, 15 min; Biofuge, Beckman). The stable radical 2,2-diphenyl-1-picrylhydrazyl (DPPH assay) was applied to assess the radical-scavenging activity of the samples (31). The analysis was performed in microplates. Fifteen microliters of extract were supplemented with 300 μl of 6- × -10–5 M methanol-DPPH. The absorbance was measured at λ = 517 nm (Cary 50 MPR Varian-Agilent). The EC50 indicated the amount of the sample (as mg) required to inhibit, after 60 min of incubation, the activity of 1 ml of the DPPH by 50%.

#### 2,20-Azino-Bis (3-Ethylbenzothiazoline-6-Sulfonic Acid) Test

The samples were soaked in methanol (1:3 w/v, plus acetic acid, 1%) and maintained overnight at 4°C (28). The supernatants were recovered (11,600 g, 15 min; Biofuge, Beckman). The ABTS test was conducted using 6-hydroxy 2,5,7,8-tetramethylchroman-2-carboxylic acid (Trolox) (32). Trolox (2.5 mM, Sigma Aldrich, Milano, Italy) was prepared in methanol. The solution of ABTS was dissolved in distilled water (final concentration, 7 mM), and a solution of potassium persulfate was also prepared (final concentration, 2.45 mM). The two solutions were combined, and the resulting mixture was kept for 16 h in the dark at room temperature before use to produce ABTS radicals (ABTS·+). The ABTS radical solution was diluted with distilled water to absorb 1.00 at λ = 734 nm. The samples (final concentrations 0.0001–0.01 mg/ml) or Trolox standards (final concentration, 0–20 mM) were added to the diluted ABTS·+ solution; we measured the absorbance 6 min after mixing (Varian). The results are expressed in μM Trolox eq (TE)/gr of a product.

### Statistical Analysis

Data are expressed as the mean ± standard deviation of triplicate measurements. Calculations were made through the PC software “Excel Statistics.” The interrelationships between the biochemical parameters and the different varieties were determined using the software package MATLAB (28, 33).

### Volatile Organic Compounds Analysis

#### Sample Preparation and HS SPME Procedure

The optimisation of the headspace (HS) SPME parameters was achieved by analysing commercial samples of apricots purchased at a local supermarket. The analysis of volatile organic compounds was accomplished according to the procedure described by Xi et al. (34), utilizing DVB/CAR/PDMS (50/30 mm) fiber at 45°C and 20 min as the extraction temperature and extraction time, respectively. For the sample preparation, 1 g of each apricot variety and 0.5 g of NaCl were placed into a 20-ml screw-on cap HS vial (Supelco, Bellefonte, PA, USA). Apricot samples were obtained by chopping several fruits and taking 1 g from the whole sample to obtain a representative sample from each cultivar. To ensure analytical reproducibility, 1 μl from a stock solution of 10 ppm of 3-octanol, used as internal standard (IS), was added to each sample. The vials were wrapped with a Teflon (PTFE) septum and an aluminium cap (Chromacol, Hertfordshire, UK) and mixed. The subsequent extraction and injection processes were automatically performed using an autosampler MPS 2 (Gerstel, Mülheim, Germany). All samples were analyzed in triplicate. The fiber was automatically introduced into the vial's septum for 20 min to permit adsorption of the volatile organic compounds onto the fiber surface. The HS-SPME fiber was preconditioned according to the manufacturer's recommendations but below the maximum advised temperature before its first use. To avoid any carryover, prior to the first daily analysis, the fiber was conditioned for 5 min at the operating temperature of the GC injector port to test the blank level.

#### Gas Chromatography–Quadrupole Mass Spectrometry Analysis (GC–QMS)

Volatile organic compounds were thermally desorbed for 10 min, and the HS SPME fiber was injected into the injector port of the gas chromatograph instrument, model GC 7890A, Agilent (Agilent Technologies, Santa Clara, USA), coupled to the mass spectrometer 5975 C (Agilent). The extracted volatile organic compounds were immediately transported to an HP-Innowax capillary column (30 m ×0.25 mm ×0.5 μm Agilent J&W) for separation. The oven temperature programme was as follows: 40°C for 2 min, increased to 160°C at 5°C min−1, increased to 250°C at 10°C min−1 and held at 250°C for 2 min. The temperatures of the ion source and quadrupole were 230°C and 150°C, respectively; helium, with a flow of 1.5 ml min−1, was the carrier gas; the injector temperature was 240°C, and the analysis was conducted in a splitless mode. Mass spectra were acquired at an ionisation energy of 70 eV, and VOCs were detected by the mass selective detector. VOC identification was achieved by matching the mass spectra with those reported by the NIST05/Wiley07 library by matching the retention indices (RI) (as Kovats indices) with literature data and from authentic commercial standards, when available. Each sample was analyzed in triplicate with a randomised sequence in which blanks were also run. A total ion chromatogram (TIC) allowed us to determine the areas of the identified VOCs; the semiquantitative data of each volatile organic compound (Relative Peak Area, RPA %) were calculated relative to the peak area of the internal standard.

### Statistical Data Analysis

Principal component analysis (PCA) was applied to investigate the VOC dataset. The observations in the space spanned by the score vectors were subjected to hierarchical cluster analysis (HCA) based on the Ward method and Euclidean distance. First, the optimal number of clusters was determined with the total silhouette maximised. Then, each cluster was characterized in terms of VOCs using the biplot generated by PCA. PCA and HCA were accomplished using in-house R-functions implemented by the R 4.0.4 platform (R Foundation for Statistical Computing).

## Results and Discussion

The presence of apricot in the diet supplies several essential secondary metabolites, such as polyphenols, β-carotene, and ascorbic acid, and it is also a source of antioxidants. Therefore, we measured the content of these metabolites and the antioxidant activity of ten traditional varieties of apricots in the Vesuvius area. Table 1 reports the total polyphenol, ascorbic acid, and β-carotene contents and the antioxidant activity data evaluated through the DPPH and ABTS tests.

**Table 1:** Total polyphenol content (TPs), ascorbic acid content, β-carotene content, and the antioxidant activity [assessed through the 2,2-diphenyl-1-picrylhydrazyl (DPPH), and 2, 20-azino-bis (3-ethylbenzothiazoline-6-sulfonic acid) (ABTS) tests] exhibited by the varieties of apricots.

	**Total polyphenols (mg GAE/ 100 g of fresh sample, ±SD)**	**Ascorbic acid** **(mg/100 g of** **fresh** **sample,±SD)**	**β-carotene** **(mg/100 g of fresh sample, ±SD)**	**DPPH** **(EC50, mg/ml** **DPPH,** **±SD)**	**ABTS** **(μM TE/ gr of product, ±SD)**
Baracca	30.22 (±3.28)	4.375 (±0.18)	0.28 (±0.051)	14.47 (± 0.79)	1.54 (± 0.03)
Boccuccia liscia	19.16 (±0.43)	2.625 (±0.015)	0.092 (±0.002)	17.07 (±0.12)	0.79 (±0.02)
Caiana	20.63 (±0.80)	9.375 (±0.09)	0.251 (±0.08)	12.24 (±0.19)	1.20 (±0.03)
Ceccona	19.73 (±1.65)	6.25 (±0.071)	0.18 (±0.03)	17.12 (±1.02)	1.19 (±0.03)
Pellecchiella	16.59 (±2.16)	4.375 (±0.05)	0.178 (±0.003)	13.06(±0.44)	1.01 (±0.03)
Presidente	12.96 (±0.56)	8.25 (±0.082)	0.204 (±0.02)	19.09 (±2.39)	0.52 (±0.12)
Preveta bella	31.32 (±2,47)	6.875 (±0.022)	0.522 (±0.03)	10.23 (±1.78)	1.14 (±0.04)
Puscia	28.67 (±2.38)	8.125 (±0.018)	0.189 (±0.05)	9.04 (±0.25)	1.20 (±0.02)
Vitillo	10.24(±0.99)	3.625 (±0.094)	0.181(±0.05)	36.27 (±3.78)	0.47 (±0.02)
Vollese	34.04 (±1.50)	10.62 (±0.092)	0.161 (±0.03)	8.27 (±0.47)	1.76 (±0.02)

*Data are reported as average ± standard deviation (SD), giving rise from the three independent experiments*.

### Ascorbic Acid Content

Ascorbic acid is an essential factor both in terms of its positive effect on human health and for food application because it has an important function in impeding the activity of enzymes such as polyphenol oxidases, thus slowing the browning of the fruits (35). The amount of ascorbic acid in the varieties of apricot that we considered for our research differed between 2.625 mg (“Boccuccia Liscia”) and 10.625 mg in 100 g of the fresh product (“Vollese”). Some varieties (“Vollese,” “Caiana,” “Presidente,” and “Puscia”) contained an amount of ascorbic acid close to those exhibited by other varieties analyzed by Ishaq et al. (35) and Fratianni et al. (28). The ascorbic acid content was superior to the values found by Cui et al. (36); in most cases, it was also more abundant than the values indicated by Wani et al. (37) and Kafkaledou et al. (38). All varieties had an ascorbic acid content similar to that found by Hegedus et al. (39), although less than or, in some cases, just slightly less (“Vollese,” “Caiana”) than that (11.5 mg 100 g-1) reported by Akin et al. (8) regarding 11 Turkish apricot varieties. With respect to the results obtained by Fratianni et al. (28), who also analyzed the varieties “Baracca” and “Boccuccia liscia,” we observed that the two cultivars showed a more or less different content of ascorbic acid. This difference, in our opinion, could be attributed not to genetic factors but to the year of harvest and the territory of origin. Therefore, these aspects could influence the compositional aspect (40). Notably, our samples were collected in 2017, and the varieties analyzed by Fratianni et al. (28) were, instead, collected in 2011 and cultivated in the western area of the Sele River (41), which is very geomorphologically different from the volcanic Vesuvius area, the origin of our samples (42).

### β-Carotene Content

The nutritional value of carotenoids is generally correlated with their aptitude to be the precursor of retinol, vitamin A, an essential nutrient for humans that cannot be synthesized within the body. This peculiarity is particular to β-carotene, which significantly supports our body. Moreover, vitamin A represents a potent gene regulator, controlling the expression of almost 700 genes. Furthermore, in the form of retinal, vitamin A is essential in mammalian vision and is required for vision, as retinal is the visual chromophore in mammals (43). A high intake of β-carotene-rich foods may play an essential role in defending telomeres by reducing oxidative stress and decreasing the risk of Alzheimer's disease (AD). It can regulate telomerase activity in old age. Additionally, lower levels of plasma β-carotene, connected to peripheral telomerase activity, are linked to AD diagnosis independent of multiple covariates (44). Apricot fruits are rich in β-carotene, which constitutes ~60–70% of the total carotenoid content (45–47) and gives the fruit its distinctive orange color. Apricot and/or β-carotene treatment is believed to be effective for preventing the impairments caused by oxidative stress, intestinal damage, and nephrotoxicity (48). Therefore, identifying apricot genotypes with high levels of β-carotene in the fruit is an important goal for both breeders and consumers. The content of β-carotene found in our samples ranged between 0.092 mg 100 g^−1^ of the fresh product (“Boccuccia liscia”) and 0.522 mg 100 g^−1^ of a fresh product (“Preveta bella”) (Table 1). This result was absolutely aligned with the values indicated by the Italian Council for Agricultural Research and Analysis of Agricultural Economics (49). “Boccuccia liscia,” as already observed by Fratianni et al. (28), once again showed the lowest amount of β-carotene, and it was inferior, considering the study performed by Leccese et al. (50), although this variety was cultivated in a different territory and collected in 2010. Therefore, the study performed by Kafkaletou et al. (38) underlined the importance of the origin on biochemistry and nutritional value of the apricots. Our results confirmed once more that the geography, soil morphology, and year of collection (affected by climate) change particular fruit and vegetable metabolic pathways (28, 50–52). The content of β-carotene was higher than that reported by Wani et al. (53), who found that the content of such molecules ranged between 156.1 and 176.7 mg kg^−1^ of dried weight. On the other hand, the same study highlighted the importance of consuming apricots as fresh products and concurrently suggested canning, freezing, and drying as methods to preserve β-carotene until four months of storage. Thus, considering the results of that paper, some of the varieties we analyzed, if consumed as fresh products or processed by canning, freezing, or drying, could support our body with a significant amount of β-carotene.

### Total Polyphenol Content

Polyphenols represent a crucial source for the antioxidant activity exhibited by fruits and vegetables. They are crucial for plants and are implicated in those mechanisms, which help them defend themselves from biotic and abiotic stresses, from attacks by pathogenic microorganisms and insects, and against environmental and climatic conditions (temperature, drought, UV radiation, salinity, the presence of heavy metals, etc.) that could, otherwise, damage their tissues in a much more incisive way (54). For human health, one of the essential functions of polyphenols can certainly be ascribable to their antioxidant activity (55). Thus, if, on the one hand, the carotenoids are quite stable, albeit in a limited time, as we have seen from the data published by Wani et al. (53), at the same time, the polyphenols could be susceptible to transformations and metabolic processes that give rise over time to other molecules. Therefore, also in this case, it is essential to identify varieties that provide a high polyphenol content. The quantity of total polyphenols (TPs), indeed, fluctuated among the varieties analyzed (Table 1). The variety “Vitillo” showed the lowest levels of TPs (10.24-mg 100-g^−1^ FW). On the other hand, other varieties exhibited a level of TPs similar or more than three times higher relative to “Vitillo,” with values ranging from 28.67-mg 100-g^−1^ FW (“Puscia”) to 30.22-mg 100-g^−1^ FW (“Baracca”) and 31.32-mg 100-g^−1^ FW (“Preveta bella”) to 34.04-mg 100-g^−1^ FW (“Vollese”). This last variety exhibited a TP content very similar to that found in the variety “Boccucia Eboli” (28). In any case, except for the variety “Vitillo,” none of the varieties we analyzed showed a total polyphenol content lower than 12-mg 100-g^−1^ FW^−1^. The varieties “Boccuccia liscia” and “Baracca” exhibited TP contents equal to 19.16-mg 100-g^−1^ FW and 30.22-mg 100-g^−1^ FW, respectively. These values were slightly lower than or similar to those observed by Fratianni et al. (28). Once again, the varieties of apricot cultivated in the Campania region, particularly in the Vesuvius territory, showed a higher TP content relative to that shown by the Iranian variety “Nouri,” analyzed by Nourozi et al. (56), and to that found by Kasapoglu et al. in some Turkish apricot varieties (57); however, it was similar to that of other varieties cultivated in the Mediterranean area, such as “Nereis” (58).

### Antioxidant Activity

**Figure 1:**
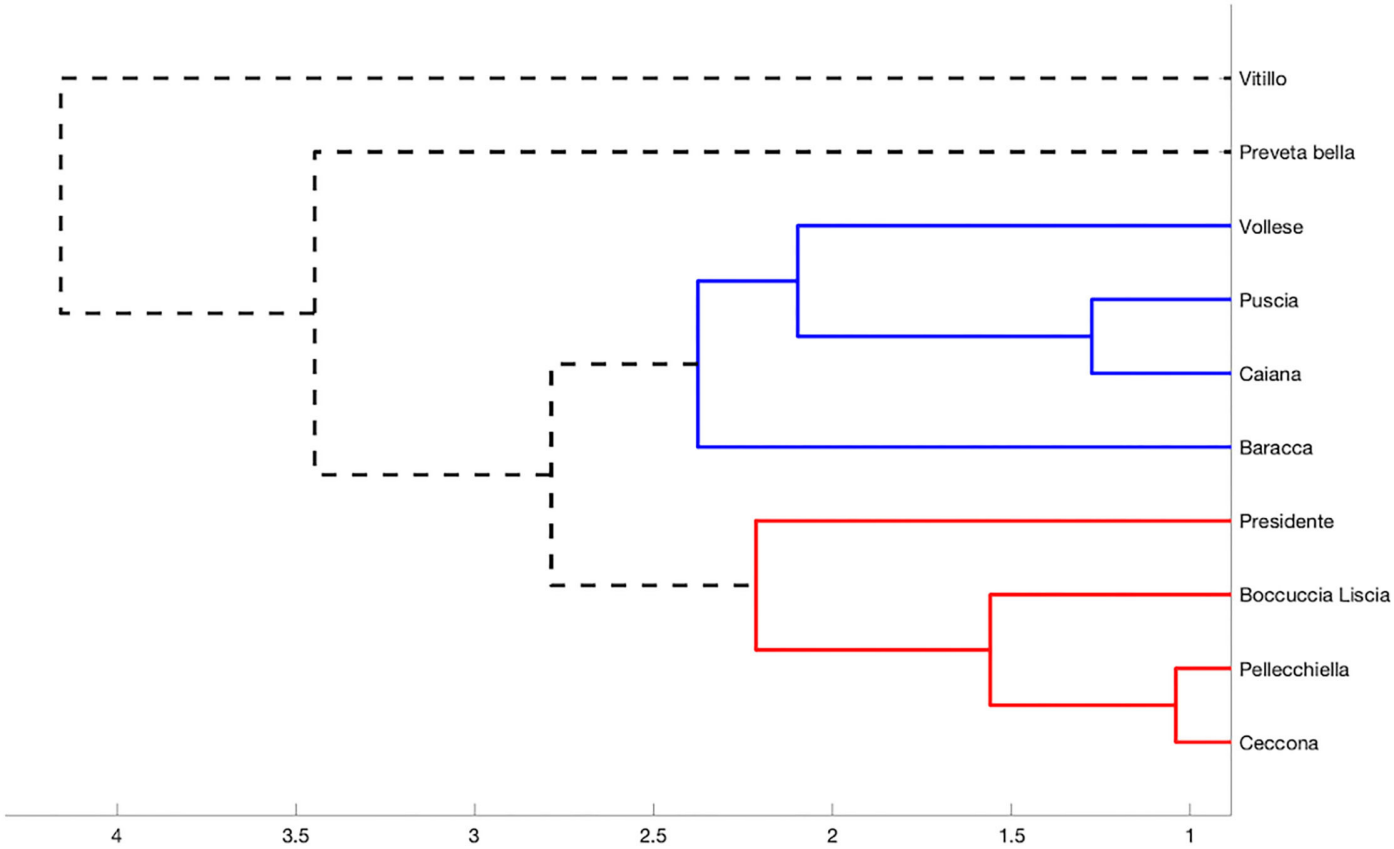
Hierarchical clustering of varieties acquired through a Euclidean unweighted distance and taking into account the normalized content of total polyphenols, ascorbic acid content, β-carotene, and antioxidant activity. We used 2.5 as the cutoff distance.

DPPH and ABTS radical scavenging capacity assays were used to obtain a broader picture of the antioxidant activity of the apricot varieties. The data are shown in Table 1. The DPPH test allowed us to quantify the reducing capacity of the samples under assessment, but, generally, the limits of this analytic approach are due to sterically large molecules, which are incapable of reacting with the responsive part of the radical. The ABTS test allowed us to evaluate and measure both the hydrophilic and lipophilic antioxidants present in our samples throughout a wide range of pH values. The DPPH radical-scavenging activity was expressed in EC50 and indicated the amount (mg) of the extract necessary for the inhibition by 50% of 1 ml of the DPPH radical molecule. The varieties exhibited EC50 values not higher than 19.09 mg ml^−1^, except “Vitillo,” which demonstrated the weakest antioxidant activity with an EC50 equal to 36.07 mg ml^−1^. The EC50 value of some of the varieties analyzed was similar to that of other varieties of the Campania region, such as “Panzona,” “Paolona,” “Puzo,” “Pazza,” and “Boccuccia spinosa”(28); although most of the other Campania varieties demonstrated a more substantial antioxidant effect, those that we analyzed showed a superior antioxidant activity with respect to some commercial dried apricots (59). The ABTS test corroborated the results of the DPPH test (ρ ABTS-DPPH = −0.73). The variety “Vollese,” which exhibited the best DPPH radical-scavenging activity (EC50 = 8.27 mg ml^−1^), also gave the best antioxidative performance with the ABTS test (1.76 μmol Trolox eq gr of a fresh product^−1^). “Puscia” and “Preveta bella” (EC50 = 9.04 and 10.23 mg ml^−1^, respectively) also gave good results with the ABTS test. The variety “Baracca,” which was less effective in inhibiting the activity of 1-ml DPPH at 50%, was, however, capable of showing excellent antioxidant activity with the ABTS test. In each case, these varieties, “Vollese,” “Puscia,” “Preveta bella,” and “Baracca,” were capable of exerting the best antioxidant activity. The analysis of correlation, performed among total polyphenol content, ascorbic acid content, β-carotene content, and antioxidant activity (evaluated through the DPPH and ABTS tests), allowed us to ascertain that the 10 varieties of apricots showed a high correlation between total polyphenols and antioxidant activity, with ρ = −0.762 and ρ = 0.875 when we considered the DPPH and ABTS tests, respectively. Such results agreed with Djenidi et al. (60) and Ishiwata et al. (61). Ascorbic acid seemed to less strongly affect the antioxidant activity (ρ = 0.538 and ρ = 0.453 for the DPPH and ABTS tests, respectively). β-carotene resulted from the weakest influencing agent on the antioxidant activity, so, from the correlation analysis, we obtained ρ = −0.256 and ρ = 0.170 (DPPH and ABTS, respectively). Most of the autochthonous apricot varieties of the Campania region are probably affected regarding the antioxidant activity by the quantity of total polyphenols they contain and, to a lesser extent, by the quantity of ascorbic acid. Again, as mentioned earlier, the antioxidant activity was not greatly affected by β-carotene. Therefore, Fratianni et al. (28) conducted comparative analyses on 24 varieties of native apricots grown in the Sele area of the Campania region (including two varieties, “Baracca” and “Boccuccia liscia,” analyzed in our work), observing the same results. Through the unweighted average Euclidean distance, the data were clustered, considering the normalized amount of total polyphenols, ascorbic acid, and β-carotene and the normalized data of the antioxidant activity. The results are shown in Figure 1. We observed that most of the varieties were clustered into two distinct groups, indicated by different colours. The group indicated by blue colour comprised four varieties: “Vollese,” “Puscia,” “Caiana,” and “Baracca.” On the other hand, the group indicated by the red colour was formed by the varieties “Ceccona,” “Pellecchiella,” “Presidente,” and “Boccuccia liscia.” The remaining varieties, “Vitillo” and “Preveta bella,” seemed to deviate quite slightly from the remaining eight varieties with respect to the biochemical parameters considered, so much so that they were quite distant from the two clusters. In the dendrogram, they are represented by a dashed black line. The hierarchical distance between the two varieties “Vitillo” and “Pellecchiella” was also observed by Leccese et al. (62). The slopes of Vesuvius are the most important Italian apricot area. After centuries of cultivation of this species, a vast population of varieties has formed, representing an interesting aspect of Italy. Additionally, from work performed on 136 varieties of the Vesuvius area, some of the varieties, including “Baracca,” “Boccuccia liscia,” “Ceccona,” and “Pellecchiella,” analyzed in our work were most interesting from an agronomic and commercial perspective when compared with other Italian and foreign varieties cultivated in three experimental fields in Rome, Caserta, and Naples (63).

### Volatile Compounds Analysis

A total of 80 volatile components were identified by HS SPME GC–MS analysis of the 10 apricot cultivars. VOCs were assembled according to the chemical classes into esters (27), terpenes (24), alcohols (10), lactones (7), aldehydes (5), ketones (3), alkanes (2), and others (2). HS SPME GC–MS semiquantitative data were subjected to one-way ANOVA to evaluate the effect of cultivar on the observed volatiles, and the results are listed in Supplementary Table 1, which also shows the abbreviation code of the VOCs, the experimental and literature-available Kovats index and the identification methods. Among the detected volatile compounds, 26 VOCs were present with different contents in all studied apricot varieties, including hexyl acetate (E15); trans-2-hexen-1-ol acetate (E18), 1-hexanol (Al4), cis-3-hexen-1-ol (Al6), 2-hexen-1-ol (Al7), 2-ethyl-1-hexanol (Al8), 1-octanol (Al9), limonene (T3), trans-β-ocimene (Al9), α-terpinolene (T9), cis linalool oxide (T10), linalool (T14), 4-terpineol (T15), β-cyclocitral (T17), α-terpineol (T19), cital (T20), geraniol (T23), geranyl acetone (T24), β-ionone (T25), hexanal (Ald2), 2-hexenal (Ald3), benzaldehyde (Ald4), 6-methylmethyl-5-hepten-2-one (K3), γ-octalactone (L2), and γ-decalactone (L5). In particular, E15, E18, Al4, Al6, Al7, T3, T14, T19, T22, Ald2, L2, and L5 were previously described as the main contributors to apricot aroma (10, 31).

**Figure 2:**
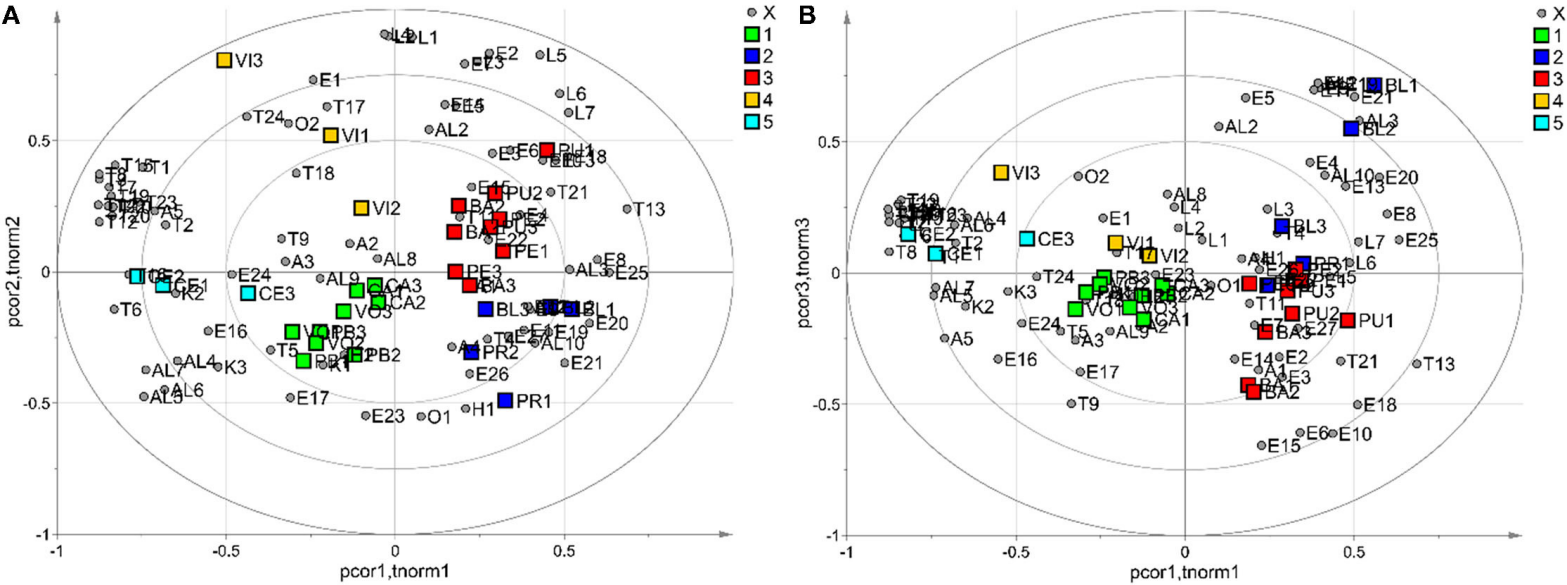
The PCA model: biplot obtained, considering the first two principal components **(A)** and biplot built, considering the first and the third principal components **(B)**; observations are coloured according to the cluster; grey circles indicate the VOCs.

Supplementary Table 1 shows that some VOCs were found in only one cultivar and could be considered cultivar-specific flavor compounds. In this regard, 2-pentanone-4-methyl (K1) and dodecane (H2) were found only in the variety “Preveta bella,” while 2-hexenyl hexanoate (E26) was exclusively present in samples of the variety “Presidente.” Ethyl octanoate (E22) was detected only in the VOC profile of the variety “Pellecchiella,” while butyl isobutyrate (E9), butyl butyrate (E12), and isobutyl alcohol (Al1) were observed only in “Boccuccia liscia.” Terpenes were the most abundant VOCs in “Preveta bella” and “Ceccona” apricots, accounting for 34.5 and 44.8% of the total volatile compounds, respectively. Among terpenes, linalool (T14) was the main constituent, representing 23.3% of the total volatiles in “Preveta bella” and 28.1% in “Ceccona.” This acyclic monoterpene alcohol, described by a floral, lavender-like odour, has also been described as the principal terpene in the apricot cultivars studied by Xi et al. (34). Ester compounds commonly observed in fruits give pleasant floral and fruity flavor attributes. In this study, esters were the dominant VOCs in seven apricot varieties, including Presidente (70.3%), Pellecchiella (55.4%), Baracca (77.3%), Puscia (62.6%), Caiana (32.5%), Vitillo (45.9%), and Vollese (42.9%). Among these samples, hexyl acetate (E15) was the most abundant volatile in Presidente (24.8%), Pellecchiella (20.1%), Puscia (27.1%), and Vollese (18.7%), while butyl acetate (E6) was the principal volatile in the Baracca cultivar. E16 and E15, defined among the major contributors to apricot aroma, were reported to be the main ester compounds in several apricot cultivars harvested in south-eastern Romania, in the southern Xinjiang region of China and in different regions of Turkey (15, 64–66). The volatile pattern of the variety “Caiana,” presenting esters as major components (32.5%), was dominated by 2-hexenal (Ald3). This compound, responsible for the herbaceous odour in many fruits, has been found to be the major C6 aldehydic compound in several apricot cultivars (16). On the other hand, the main volatile component of “Vitillo” was linalool (T14), accounting for 21.2% of the total volatiles. Alcohols were the predominant VOCs in “Boccuccia liscia” (31.8%), with 1-hexanol (Al4) as the most abundant compound (21.7%) of the total volatiles. Al4, produced by the LOX pathway and showing a characteristic odour described as green, grassy, and leafy, was the main alcohol in several apricot cultivars (16). The composition and amount of VOCs presented significant differences among the ten apricot varieties, as the variety could strongly affect the profile of volatile components, which, in turn, have been revealed to be extremely valuable to identify different fruit origins (genetic or geographic) (66, 67). For this reason, an exploratory data analysis performed by PCA was applied to evaluate the effectiveness of VOC profiles to explain possible variations in the volatile content dependent on the genotype and identify putative volatile markers suitable for cultivar differentiation. The dataset comprised 29 observations (9 biological samples as technical triplicates and one biological sample as a technical duplicate), and 80 VOCs were explored by PCA. Data were autoscaled before the data analysis was performed. The PCA model showed 3 principal components with R2 = 0.55 and Q2 = 0.28 (calculated by 5-fold cross-validation based on the Krzanowski method), and HCA generated 5 clusters. Specifically, the first cluster was related to “Preveta bella,” “Caiana,” and “Vollese” (Cluster 1), the second cluster was related to “Presidente” and “Boccuccia liscia” (Cluster 2), and the third cluster was related to “Pellecchiella,” “Baracca,” and “Puscia” (Cluster 3). In contrast, the last two clusters referred to “Vitillo” (Cluster 4) and “Ceccona” (Cluster 5), respectively. In Figure 2, displaying the biplots of the PCA, the samples are coloured according to the cluster. The investigation of the biplots allowed the characterisation of every single cluster in terms of VOCs. In detail, Clusters 3 and 4 showed the highest amount of E2, E7, L1, L2, L3, L4, and L5, agreeing with the data listed in Supplementary Table 1. In particular, propyl acetate (E2) (fruity odour), present only in the samples belonging to Clusters 3 and 4, has been reported among the primary compounds responsible for the aroma in some apricot varieties (9). Regarding the lactones, L1, L2, L3, L4, and L5 were detected in all of the samples involved in Clusters 3 and 4 (“Pellecchiella,” “Baracca,” “Puscia,” and “Vitillo”), with γ-decalactone (L5) being the most abundant in all four apricot varieties. Lactones have been believed to be responsible for the typical sweet and fruity sensory properties of fresh apricots and are considered key characteristic flavor factors contributing to apricot consumer acceptance (16, 64). The most abundant volatile compounds discriminating apricot varieties contained in Clusters 2 and 3 from the other groups were E8, E25, and T13 (Figure 2). These three volatiles were detected together only in the five varieties (“Presidente,” “Boccuccia liscia,” “Pellecchiella,” “Baracca,” and “Puscia”) included in these two clusters. Specifically, butyl propionate (E8), with a fruity odour, and hexyl hexanoate (E25), characterized by a fruity with berry note smell, have been previously reported in other apricot varieties (16, 68). Theaspirane, commonly detected in raspberry and wild cherry, has been reported to show wood- and menthol-like odours (69). In earlier reports, aspirin was rarely identified in apricots, and its production was believed to be related to the presence of β-ionone (60), which was also detected in our study. Figure 2 shows that 15 terpenes, including T1, T2, T3, T6, T7, T8, T10, T12, T14, T15, T16, T19, T20, T22, and T23, were the main contributors to Cluster 5, while the lowest levels of these compounds were associated with Clusters 2 and 3, consistent with the results presented in Table 2. In detail, as already mentioned above, the VOC profile of “Ceccona,” forming Cluster 5, was dominated by the terpene components, with linalool (T14) and α-terpineol (T19) (28.1 and 11.2% of the total VOC content, respectively) as the major metabolites. Terpenes, described by herbaceous, fruity, citrus, and floral scents, are recognised for their contribution to the pleasant sensory odours of fruit (67). Distinctly, terpene alcohols, such as linalool and α-terpineol, have been designated contributors to the floral and fruity notes of several apricot cultivars (70). As indicated in the Materials and Methods section, the semiquantitative data of each volatile (relative peak area, RPA %) were calculated relative to the peak area of 3-octanol used as the internal standard to ensure analytical reproducibility. The majority of the varieties analyzed, particularly “Puscia,” “Baracca,” “Caiana,” “Pellecchiella,” and “Vollese,” showed a total RPA %, ranging between 96.53 (Puscia) and 186.72 (Caiana). Vitillo and Ceccona exhibited the most abundant content of total terpenes, particularly Ceccona (total RPA % = 656.48), with a terpene content ~14 times higher than those present in the varieties Boccuccia liscia and Presidente (RPA % = 47.09 and 49.77, respectively). Although the antioxidant activity of terpenes has been reported in the literature (71), these molecules (identifiable in Supplementary Material Table 1 as T1-T25 compounds) did not appear to have influenced such activity. The correlation analysis performed between total terpenes and the antioxidant activity did not yield any positive results. α-Terpinolene was the only terpene that seemed to affect the antioxidant activity in the DPPH test (ρ = −0.29) and, above all, (ρ = 0.78) in the ABTS test. Such results seemed to confirm the antioxidant action exerted by this molecule found in other plant derivatives, especially Melaleuca alternifolia oil (72, 73). In contrast to other studies that evidenced their antioxidant activity (74–76), the two most abundant molecules, linalool (ρ = 0.43 and ρ = −0.10, for DPPH and ABTS, respectively) and α-terpineol (ρ = 0.46 and ρ = 0.12, for DPPH and ABTS, respectively) seemed to possess an inhibitory effect on the antioxidant activity, such that, from the correlation analysis made with respect to total % RPA, the correlation values resulted in very similar ρ = 0.43 and ρ = −0.06, for DPPH and ABTS, respectively.

Finally, four alcohols, including Al4, Al5, Al6, and Al7, were the prominent aroma compounds measured in the samples belonging to Clusters 1 and 5, whereas they were the lowest metabolites detected in the samples included in Cluster 3. Alcohols mainly contribute to the flowery, green, and herbaceous odours in different fruits; consistent with early studies, they have previously been described as pertinent flavor volatiles in apricots (13, 64). In line with the data reported in Table 2, we detected Al4, Al5, Al6, and Al7 in all of the apricot varieties belonging to Clusters 1 and 5. Among the alcohol groups, 1-hexanol (Al4) (5.2–10.8%) and trans-2-hexen-1-ol (Al7) (7–15.8%), which originate from lipoxygenase activity and are involved in the cleavage of unsaturated fatty acids, were the main alcohols identified in all the samples of both clusters. The biplots of the PCA performed on the volatile contents allowed us to discriminate the 10 varieties in five different clusters in terms of VOCs. According to our data, the VOC profiles of the varieties “Pellecchiella,” “Baracca,” “Puscia,” and “Vitillo” (Clusters 3 and 4), which were dominated by two acetate esters and five lactones, seem to be mainly characterized by sweet and fruity notes. Furthermore, apricots belonging to Cluster 3 together with samples of Cluster 2 (“Presidente” and “Boccuccia liscia”), being dominated by the esters E8 and E25 and T13, can be described by berry-like fruity odours. Fifteen terpenes were the main contributors to Cluster 5, comprising “Ceccona” apricots, suggesting that this cultivar could mainly be designated by floral smell. Finally, the higher contents of alcohols detected in Clusters 1 and 5 (varieties “Preveta bella,” “Caiana,” “Vollese,” and “Ceccona”) probably enhance the green notes in these varieties. Further studies should be performed to confirm these hypotheses by evaluating the sensory parameters of the apricot varieties through the use of a panel test comprising trained judges to verify whether the presence of the different VOCs discriminating the varieties can also affect their herbaceous and/or fruit notes from a sensory perspective.

Several papers have described different quality aspects of a wide number of apricot ecotypes of the Campania region, including their inter- and intra-variability from a morphological and genetic point of view and the comprehensive characterisation of the different phytochemicals, as mentioned in the introduction section, present in this fruit (22–28). On the other hand, in the present study, for the first time, to the authors' knowledge, the concurrent biochemical and sensory parameters of ten apricots landraces produced in the niche geographical Vesuvius area have been evaluated.

Overall, our findings, besides providing consumers with enhanced knowledge of health properties of these apricot landraces, could contribute to define and preserve a precious genetic and cultural-historical biodiversity heritage, which are at risk of extinction, owing to expanding urbanization, intensification farming, and renewing varieties.

## Conclusions

Protection of plant biodiversity is an objective to be pursued for the well-being of the consumer, the conservation and enhancement of the agro-food traditions of a specific territory, and increase in the economic income of the operators in the sector. The biochemical characterisation of the varieties of apricots native to the Vesuvius area and witnesses of the millenary history of *Campaniafelix* highlighted their nutritional potential, demonstrating for some of them a good content of nutraceutical elements that are important for human health. Furthermore, our study conducted on total polyphenols, ascorbic acid, and β-carotene, as well as on antioxidant activity, could determine an increase in the cultivation of apricots to ensure their consumption not only as a fresh product but also in the form of juices, nectars, and jams. Finally, for the first time, at least to our knowledge, these varieties were also characterized for their VOC profile. Our results demonstrated a significant effect of the varieties on the VOC profiles, both from a qualitative and semiquantitative perspective, classifying the varieties into different clusters, each characterized by specific notes. This work can form the basis for further research on the Vesuvius apricots, worthy of being valued not only for their quality but also for their nutritional properties so as to increase their cultivation.

## Data Availability Statement

The original contributions presented in the study are included in the article/Supplementary Material, further inquiries can be directed to the corresponding author.

## Author Contributions

FN and FF: conceptualization. FF and RC: methodology. Ad'A, RC, LM, and MS: software. FF, RC, FN, and MO: investigation. PS and RR: resources. FN, RC, and FF: writing—original draft preparation. FN, RC, FF, and Ad'A: writing—review and editing. FN: supervision. FN, PS, and RR: funding acquisition. All the authors have read and agreed to the published version of the manuscript.

## Funding

This research was funded by the project IPark – Presidio e Cittadinanza supported by the Italian Foundation Fondazione con il Sud-Bando Ambiente Project code 2015-AMB-0070.

## Conflict of Interest

The authors declare that the research was conducted in the absence of any commercial or financial relationships that could be construed as a potential conflict of interest.

## Publisher's Note

All claims expressed in this article are solely those of the authors and do not necessarily represent those of their affiliated organizations, or those of the publisher, the editors and the reviewers. Any product that may be evaluated in this article, or claim that may be made by its manufacturer, is not guaranteed or endorsed by the publisher.
