# Supplementary material for: Biochemical Characterization of Some Varieties of Apricot Present in the Vesuvius Area, Southern Italy
Source: Front Nutr. 2022 Mar 8;9:854868. doi: 10.3389/fnut.2022.854868 (PMC8958034; doi:10.3389/fnut.2022.854868)
Supplement: Supplementary file 1 [file Table_1.docx]

**Table 1.** Volatile compounds detected the in the ten apricot cultivars and their identification codes

| **Metabolites** | **Code** | **RIcal/ RIt** | | | **ID** | |  | | | | | | | | | | | | | | | | | | | | | | | | | | | | | | | | | | | | | | | | | | | | | | | | | | | | |  |  |
| --- | --- | --- | --- | --- | --- | --- | --- | --- | --- | --- | --- | --- | --- | --- | --- | --- | --- | --- | --- | --- | --- | --- | --- | --- | --- | --- | --- | --- | --- | --- | --- | --- | --- | --- | --- | --- | --- | --- | --- | --- | --- | --- | --- | --- | --- | --- | --- | --- | --- | --- | --- | --- | --- | --- | --- | --- | --- | --- | --- | --- | --- |
|  |  |  | | |  | | ***Prevete Bella*** | | | | ***Presidente*** | | | ***Pellecchiella*** | | | | ***Baracca*** | | | | ***Boccuccia***  ***liscia*** | | | | | ***Puscia*** | | | | | ***Caiana*** | | | | | ***Vitillo*** | | | | | ***Ceccona*** | | | | | | | | ***Vollese*** | | | | | | | | ***P*** | | |  |
| Isobutyl alcohol | AL1 | 1401/1400 | RI/MS | 0.00 | | **a** | | 0,00 | | **a** | | 0,00 | | **a** | 0,00 | | **a** | | 1,38 | | **b** | | 0,00 | | **a** | | | 0,00 | | | **a** | | | 0,00 | | | **a** | | 0,00 | | | | **a** | | | 0,00 | | | **a** | | | | ******* | | |  |  |  |  |  |  |
| Isoamyl alcohol | AL2 | 1115/1113 | RI/MS | 0.00 | | **a** | | 0,39 | | **a** | | 0,00 | | **a** | 0,00 | | **a** | | 3,05 | | **a** | | 2,57 | | **a** | | | 0,90 | | | **a** | | | 4,09 | | | **a** | | 0,00 | | | | **a** | | | 0,00 | | | **a** | | | | ***** | | |  |  |  |  |  |  |
| 2-Buten-1-ol-3-methyl | AL3 | 1231/1237 | RI/MS | 0.00 | | **a** | | 0,00 | | **a** | | 0,00 | | **a** | 0,00 | | **a** | | 0,44 | | **b** | | 0,17 | | **ab** | | | 0,00 | | | **a** | | | 0,00 | | | **a** | | 0,00 | | | | **a** | | | 0,00 | | | **a** | | | | ******* | | |  |  |  |  |  |  |
| 1-Hexanol | AL4 | 1336/1337 | RI/MS | 67.7 | | **abc** | | 8,59 | | **a** | | 27,07 | | **a** | 11,53 | | **a** | | 67,79 | | **abc** | | 30,90 | | **ab** | | | 40,83 | | | **ab** | | | 17,31 | | | **a** | | 146,54 | | | | **c** | | | 113,96 | | | **bc** | | | | ******* | | |  |  |  |  |  |  |
| *trans*-3-Hexen-1-ol | AL5 | 1367/1366 | RI/MS | 1.62 | | **b** | | 0,00 | | **a** | | 0,00 | | **a** | 0,00 | | **a** | | 0,00 | | **a** | | 0,00 | | **a** | | | 0,77 | | | **ab** | | | 0,00 | | | **a** | | 1,64 | | | | **b** | | | 1,68 | | | **b** | | | | ******* | | |  |  |  |  |  |  |
| *cis*-3-Hexen-1-ol | AL6 | 1374/1374 | RI/MS | 26.21 | | **b** | | 0,46 | | **a** | | 4,96 | | **a** | 0,40 | | **a** | | 11,39 | | **ab** | | 1,75 | | **a** | | | 11,87 | | | **ab** | | | 2,53 | | | **a** | | 28,98 | | | | **b** | | | 15,83 | | | **ab** | | | | ******* | | |  |  |  |  |  |  |
| 2-Hexen-1-ol | AL7 | 1394/1394 | RI/MS | 89.96 | | **abc** | | 6,29 | | **a** | | 9,12 | | **a** | 2,25 | | **a** | | 10,30 | | **a** | | 16,82 | | **a** | | | 54,33 | | | **ab** | | | 21,68 | | | **a** | | 134,49 | | | | **bc** | | | 167,59 | | | **c** | | | | ******* | | |  |  |  |  |  |  |
| 1-Hexanol-2-ethyl | AL8 | 1412/1410 | RI/MS | 1.83 | | **a** | | 3,77 | | **ab** | | 1,48 | | **a** | 2,37 | | **ab** | | 3,01 | | **ab** | | 2,13 | | **ab** | | | 2,52 | | | **ab** | | | 2,98 | | | **ab** | | 2,71 | | | | **ab** | | | 2,51 | | | **ab** | | | | ***** | | |  |  |  |  |  |  |
| 1-Octanol | AL9 | 1498/1499 | RI/MS | 0,43 | |  | | 0,44 | |  | | 0,50 | |  | 0,63 | |  | | 0,51 | |  | | 0,37 | |  | | | 0,58 | | |  | | | 0,44 | | |  | | 0,64 | | | |  | | | 0,66 | | |  | | | | **ns** | | |  |  |  |  |  |  |
| Benzyl alcohol | AL10 | 1561/1565 | RI/MS | 0,00 | | **a** | | 0,00 | | **a** | | 1,45 | | **a** | 0,00 | | **a** | | 1,47 | | **a** | | 0,00 | | **a** | | | 0,00 | | | **a** | | | 0,00 | | | **a** | | 0,00 | | | | **a** | | | 0,53 | | | **a** | | | | ***** | | |  |  |  |  |  |  |
| Ethyl acetate | E1 | 869/870 | RI/MS | 0,35 | | **a** | | 3,51 | | **a** | | 26,15 | | **a** | 32,03 | | **a** | | 0,00 | | **a** | | 37,97 | | **a** | | | 35,90 | | | **a** | | | 370,32 | | | **b** | | 16,22 | | | | **a** | | | 0,00 | | | **a** | | | | ******* | | |  |  |  |  |  |  |
| n-Propyl acetate | E2 | 974/974 | RI/MS | 0,00 | | **a** | | 0,00 | | **a** | | 4,06 | | **ab** | 7,34 | | **b** | | 0,00 | | **a** | | 7,27 | | **b** | | | 0,00 | | | **a** | | | 7,02 | | | **b** | | 0,00 | | | | **a** | | | 0,00 | | | **a** | | | | ******* | | |  |  |  |  |  |  |
| Isobutyl acetate | E3 | 1020/1020 | RI/MS | 0,00 | | **a** | | 1,28 | | **a** | | 1,57 | | **a** | 25,64 | | **a** | | 0,00 | | **a** | | 34,72 | | **a** | | | 1,18 | | | **a** | | | 6,47 | | | **a** | | 0,00 | | | | **a** | | | 0,00 | | | **a** | | | | ***** | | |  |  |  |  |  |  |
| Ethyl butanoate | E4 | 1043/1044 | RI/MS | 0,00 | | **a** | | 0,70 | | **ab** | | 4,49 | | **b** | 0,00 | | **a** | | 2,26 | | **ab** | | 0,97 | | **ab** | | | 0,00 | | | **a** | | | 1,14 | | | **ab** | | 1,07 | | | | **ab** | | | 0,00 | | | **a** | | | | ******* | | |  |  |  |  |  |  |
| Ethyl 2-methylbutanoate | E5 | 1056/1056 | RI/MS | 0,00 | | **a** | | 0,00 | | **a** | | 0,47 | | **ab** | 0,00 | | **a** | | 0,81 | | **ab** | | 0,39 | | **ab** | | | 0,00 | | | **a** | | | 1,05 | | | **ab** | | 0,00 | | | | **a** | | | 0,00 | | | **a** | | | | ***** | | |  |  |  |  |  |  |
| Butyl acetate | E6 | 1079/1078 | RI/MS | 0,00 | | **a** | | 81,32 | | **a** | | 131,95 | | **ab** | 423,52 | | **b** | | 1,45 | | **a** | | 263,68 | | **ab** | | | 4,41 | | | **a** | | | 93,13 | | | **a** | | 33,48 | | | | **a** | | | 113,49 | | | **ab** | | | | ******* | | |  |  |  |  |  |  |
| Isoamyl acetate | E7 | 1133/1133 | RI/MS | 0,00 | | **a** | | 1,26 | | **a** | | 1,45 | | **a** | 2,63 | | **a** | | 0,00 | | **a** | | 6,72 | | **a** | | | 2,00 | | | **a** | | | 5,18 | | | **a** | | 0,00 | | | | **a** | | | 0,00 | | | **a** | | | | ******* | | |  |  |  |  |  |  |
| Butyl propionate | E8 | 1156/1153 | RI/MS | 0,00 | | **a** | | 1,31 | | **ab** | | 1,69 | | **ab** | 1,08 | | **ab** | | 2,33 | | **b** | | 2,49 | | **b** | | | 0,00 | | | **a** | | | 0,00 | | | **a** | | 0,87 | | | | **ab** | | | 1,30 | | | **ab** | | | | ****** | | |  |  |  |  |  |  |
| Butyl isobutyrate | E9 | 1160/1159 | RI/MS | 0,00 | | **a** | | 0,00 | | **a** | | 0,00 | | **a** | 0,00 | | **a** | | 1,63 | | **b** | | 0,00 | | **a** | | | 0,00 | | | **a** | | | 0,00 | | | **a** | | 0,00 | | | | **a** | | | 0,00 | | | **a** | | | | ***** | | |  |  |  |  |  |  |
| Pentyl acetate | E10 | 1197/1192 | RI/MS | 0,00 | | **a** | | 5,06 | | **ab** | | 8,52 | | **ab** | 21,93 | | **b** | | 0,00 | | **a** | | 18,85 | | **ab** | | | 1,04 | | | **a** | | | 1,93 | | | **a** | | 1,49 | | | | **a** | | | 4,77 | | | **ab** | | | | ******* | | |  |  |  |  |  |  |
| Butyl butanoate | E11 | 1238/1243 | RI/MS | 0,00 | | **a** | | 3,77 | | **a** | | 3,41 | | **a** | 0,00 | | **a** | | 13,79 | | **b** | | 3,54 | | **a** | | | 0,00 | | | **a** | | | 0,00 | | | **a** | | 3,80 | | | | **a** | | | 4,99 | | | **a** | | | | ****** | | |  |  |  |  |  |  |
| Buty 2-methylbutanoate | E12 | 1252/1253 | RI/MS | 0,00 | | **a** | | 0,00 | | **a** | | 0,00 | | **a** | 0,00 | | **a** | | 1,96 | | **b** | | 0,00 | | **a** | | | 0,00 | | | **a** | | | 0,00 | | | **a** | | 0,00 | | | | **a** | | | 0,00 | | | **a** | | | | ******* | | |  |  |  |  |  |  |
| Ethyl hexanoate | E13 | 1255/1258 | RI/MS | 0,00 | | **a** | | 0,88 | | **ab** | | 3,64 | | **b** | 0,00 | | **a** | | 1,79 | | **ab** | | 1,63 | | **ab** | | | 0,00 | | | **a** | | | 1,58 | | | **ab** | | 0,00 | | | | **a** | | | 0,00 | | | **a** | | | | ******* | | |  |  |  |  |  |  |
| 2-Buten-1-ol-3-methyl acetate | E14 | 1272/1266 | RI/MS | 0,00 | | **a** | | 0,44 | | **ab** | | 0,64 | | **abc** | 1,26 | | **c** | | 0,00 | | **a** | | 0,65 | | **bc** | | | 1,03 | | | **bc** | | | 1,37 | | | **c** | | 0,00 | | | | **a** | | | 0,00 | | | **a** | | | | ******* | | |  |  |  |  |  |  |
| Hexyl acetate | E15 | 1289/1289 | RI/MS | 59,47 | | **ab** | | 153,19 | | **ab** | | 158,89 | | **ab** | 367,23 | | **b** | | 1,95 | | **a** | | 318,69 | | **b** | | | 59,35 | | | **ab** | | | 71,46 | | | **ab** | | 120,75 | | | | **ab** | | | 198,65 | | | **ab** | | | | ****** | | |  |  |  |  |  |  |
| *cis*-3-hexen-1-ol acetate | E16 | 1329/1329 | RI/MS | 87,07 | | **a** | | 42,44 | | **a** | | 64,43 | | **a** | 54,39 | | **a** | | 0,00 | | **a** | | 13,39 | | **a** | | | 75,71 | | | **a** | | | 36,60 | | | **a** | | 90,57 | | | | **a** | | | 51,00 | | | **a** | | | | ***** | | |  |  |  |  |  |  |
| *trans*-hexen-1-ol acetate | E17 | 1346/1342 | RI/MS | 62,56 | | **ab** | | 127,82 | | **b** | | 19,65 | | **a** | 53,06 | | **a** | | 0,29 | | **a** | | 22,96 | | **a** | | | 72,00 | | | **ab** | | | 27,58 | | | **a** | | 62,83 | | | | **ab** | | | 77,21 | | | **ab** | | | | ***** | | |  |  |  |  |  |  |
| Heptyl acetate | E18 | 1380/1380 | RI/MS | 0,00 | | **a** | | 0,00 | | **a** | | 0,44 | | **a** | 0,65 | | **a** | | 0,00 | | **a** | | 0,74 | | **a** | | | 0,00 | | | **a** | | | 0,00 | | | **a** | | 0,00 | | | | **a** | | | 0,00 | | | **a** | | | | ******* | | |  |  |  |  |  |  |
| Butyl hexanoate | E19 | 1416/1416 | RI/MS | 0,00 | | **a** | | 0,55 | | **ab** | | 0,00 | | **a** | 0,00 | | **a** | | 0,90 | | **b** | | 0,00 | | **a** | | | 0,00 | | | **a** | | | 0,00 | | | **a** | | 0,00 | | | | **a** | | | 0,00 | | | **a** | | | | ******* | | |  |  |  |  |  |  |
| Hexyl butyrate | E20 | 1420/1420 | RI/MS | 0,00 | | **a** | | 3,91 | | **c** | | 3,20 | | **bc** | 0,00 | | **a** | | 2,82 | | **bc** | | 1,85 | | **abc** | | | 0,00 | | | **a** | | | 0,00 | | | **a** | | 0,95 | | | | **ab** | | | 0,72 | | | **ab** | | | | ******* | | |  |  |  |  |  |  |
| Hexyl 2-methylbutanoate | E21 | 1432/1433 | RI/MS | 0,00 | | **a** | | 1,05 | | **b** | | 0,00 | | **a** | 0,00 | | **a** | | 1,49 | | **b** | | 0,00 | | **a** | | | 0,00 | | | **a** | | | 0,00 | | | **a** | | 0,00 | | | | **a** | | | 0,00 | | | **a** | | | | ******* | | |  |  |  |  |  |  |
| Ethyl octanoate | E22 | 1436/1436 | RI/MS | 0,00 | | **a** | | 0,00 | | **a** | | 0,47 | | **b** | 0,00 | | **a** | | 0,00 | | **a** | | 0,00 | | **a** | | | 0,00 | | | **a** | | | 0,00 | | | **a** | | 0,00 | | | | **a** | | | 0,00 | | | **a** | | | | ******* | | |  |  |  |  |  |  |
| *trans*-2-Hexenyl butyrate | E23 | 1479/1476 | RI/MS | 0,79 | | **a** | | 3,82 | | **b** | | 0,51 | | **a** | 0,00 | | **a** | | 0,00 | | **a** | | 0,00 | | **a** | | | 0,00 | | | **a** | | | 0,00 | | | **a** | | 0,95 | | | | **a** | | | 1,39 | | | **ab** | | | | ******* | | |  |  |  |  |  |  |
| Ethyl Nonanoate | E24 | 1537/1534 | RI/MS | 0,41 | | **a** | | 0,00 | | **a** | | 0,00 | | **a** | 0,52 | | **a** | | 0,00 | | **a** | | 0,00 | | **a** | | | 0,00 | | | **a** | | | 0,53 | | | **a** | | 0,42 | | | | **a** | | | 1,08 | | | **b** | | | | ******* | | |  |  |  |  |  |  |
| Hexyl hexanoate | E25 | 1609/1606 | RI/MS | 0,00 | | **a** | | 0,68 | | **a** | | 0,58 | | **a** | 0,00 | | **a** | | 0,30 | | **a** | | 0,43 | | **a** | | | 0,00 | | | **a** | | | 0,00 | | | **a** | | 0,00 | | | | **a** | | | 0,00 | | | **a** | | | | ****** | | |  |  |  |  |  |  |
| 2-Hexenyl hexanoate | E26 | 1671/1668 | RI/MS | 0,00 | | **a** | | 1,05 | | **b** | | 0,00 | | **a** | 0,00 | | **a** | | 0,00 | | **a** | | 0,00 | | **a** | | | 0,00 | | | **a** | | | 0,00 | | | **a** | | 0,00 | | | | **a** | | | 0,00 | | | **a** | | | | ******* | | |  |  |  |  |  |  |
| Benzyl acetate | E27 | 1730/1727 | RI/MS | 0,00 | | **a** | | 0,76 | | **b** | | 0,52 | | **ab** | 0,22 | | **ab** | | 0,00 | | **a** | | 0,00 | | **a** | | | 0,32 | | | **ab** | | | 0,00 | | | **a** | | 0,00 | | | | **a** | | | 0,00 | | | **a** | | | | ******* | | |  |  |  |  |  |  |
| β-Myrcene | T1 | 1174/1174 | RI/MS | 1,60 | | **ab** | | 0,00 | | **a** | | 1,03 | | **ab** | 1,06 | | **ab** | | 0,00 | | **a** | | 1,08 | | **ab** | | | 1,19 | | | **ab** | | | 3,31 | | | **b** | | 3,35 | | | | **b** | | | 1,76 | | | **ab** | | | | ***** | | |  |  |  |  |  |  |
| α-Terpinene | T2 | 1192/1193 | RI/MS | 0,35 | | **ab** | | 0,00 | | **a** | | 0,35 | | **ab** | 0,18 | | **a** | | 0,00 | | **a** | | 0,00 | | **a** | | | 0,00 | | | **a** | | | 0,63 | | | **ab** | | 0,95 | | | | **b** | | | 0,49 | | | **ab** | | | | ***** | | |  |  |  |  |  |  |
| dl-Limonene | T3 | 1211/1215 | RI/MS | 4,69 | | **ab** | | 0,86 | | **a** | | 2,08 | | **ab** | 1,86 | | **ab** | | 1,12 | | **a** | | 1,72 | | **ab** | | | 2,74 | | | **ab** | | | 8,14 | | | **b** | | 8,50 | | | | **b** | | | 4,03 | | | **ab** | | | | ******* | | |  |  |  |  |  |  |
| Eucalyptol | T4 | 1225/1225 | RI/MS | 0,00 | | **a** | | 0,51 | | **ab** | | 0,98 | | **b** | 0,00 | | **a** | | 0,58 | | **ab** | | 0,00 | | **a** | | | 0,75 | | | **ab** | | | 0,00 | | | **a** | | 0,00 | | | | **a** | | | 0,63 | | | **ab** | | | | ******* | | |  |  |  |  |  |  |
| *cis*-Ocimene | T5 | 1248/1250 | RI/MS | 9,29 | |  | | 1,79 | |  | | 2,84 | |  | 3,17 | |  | | 0,00 | |  | | 0,00 | |  | | | 0,00 | | |  | | | 1,57 | | |  | | 3,87 | | | |  | | | 10,20 | | |  | | | | **ns** | | |  |  |  |  |  |  |
| γ-Terpinene | T6 | 1258/1258 | RI/MS | 0,48 | | **a** | | 0,00 | | **a** | | 0,00 | | **a** | 0,00 | | **a** | | 0,00 | | **a** | | 0,00 | | **a** | | | 0,00 | | | **a** | | | 0,33 | | | **a** | | 1,55 | | | | **b** | | | 0,59 | | | **a** | | | | ******* | | |  |  |  |  |  |  |
| *trans*-β-Ocimene | T7 | 1265/1259 | RI/MS | 1,26 | | **ab** | | 0,19 | | **a** | | 0,50 | | **a** | 0,52 | | **a** | | 0,22 | | **a** | | 0,46 | | **a** | | | 1,16 | | | **ab** | | | 2,01 | | | **ab** | | 2,59 | | | | **b** | | | 0,50 | | | **a** | | | | ******* | | |  |  |  |  |  |  |
| p-Cymene | T8 | 1282/1282 | RI/MS | 0,33 | | **ab** | | 0,00 | | **a** | | 0,25 | | **ab** | 0,20 | | **ab** | | 0,00 | | **a** | | 0,14 | | **ab** | | | 0,38 | | | **ab** | | | 0,74 | | | **ab** | | 0,79 | | | | **b** | | | 0,52 | | | **ab** | | | | ******* | | |  |  |  |  |  |  |
| α-Terpinolene | T9 | 1292/1292 | RI/MS | 7,01 | | **ab** | | 0,00 | | **a** | | 9,00 | | **ab** | 18,18 | | **b** | | 0,47 | | **a** | | 12,58 | | **b** | | | 6,29 | | | **ab** | | | 7,25 | | | **ab** | | 16,97 | | | | **b** | | | 16,23 | | | **b** | | | | ***** | | |  |  |  |  |  |  |
| *cis*-Linalool oxide | T10 | 1448/1448 | RI/MS | 2,02 | | **a** | | 1,54 | | **a** | | 1,57 | | **a** | 0,51 | | **a** | | 0,42 | | **a** | | 1,11 | | **a** | | | 3,65 | | | **a** | | | 5,40 | | | **a** | | 6,37 | | | | **a** | | | 3,34 | | | **a** | | | | ******* | | |  |  |  |  |  |  |
| Dihydromyrcenol | T11 | 1475/1473 | RI/MS | 0,00 | | **a** | | 0,00 | | **a** | | 0,00 | | **a** | 0,28 | | **a** | | 0,24 | | **a** | | 0,00 | | **a** | | | 0,00 | | | **a** | | | 0,19 | | | **a** | | 0,00 | | | | **a** | | | 0,00 | | | **a** | | | | ***** | | |  |  |  |  |  |  |
| *trans*-Linalool oxide | T12 | 1475/1478 | RI/MS | 0,60 | | **abc** | | 0,00 | | **a** | | 0,00 | | **a** | 0,00 | | **a** | | 0,00 | | **a** | | 0,12 | | **ab** | | | 1,08 | | | **bc** | | | 1,24 | | | **c** | | 1,53 | | | | **c** | | | 0,65 | | | **abc** | | | | ******* | | |  |  |  |  |  |  |
| Theaspirane A/B | T13 | 1502/1500 | RI/MS | 0,00 | | **a** | | 1,38 | | **ab** | | 0,73 | | **ab** | 2,52 | | **b** | | 1,25 | | **ab** | | 2,60 | | **b** | | | 1,16 | | | **ab** | | | 0,00 | | | **a** | | 0,00 | | | | **a** | | | 0,00 | | | **a** | | | | ******* | | |  |  |  |  |  |  |
| Linalool | T14 | 1549/1549 | RI/MS | 195,11 | | **ab** | | 26,76 | | **a** | | 60,70 | | **ab** | 55,96 | | **ab** | | 26,56 | | **a** | | 47,72 | | **ab** | | | 102,26 | | | **ab** | | | 288,88 | | | **bc** | | 407,77 | | | | **c** | | | 89,84 | | | **ab** | | | | ******* | | |  |  |  |  |  |  |
| 4-Terpineol | T15 | 1604/1604 | RI/MS | 1,02 | | **ab** | | 0,31 | | **a** | | 0,96 | | **ab** | 0,41 | | **a** | | 0,34 | | **a** | | 0,71 | | **ab** | | | 1,42 | | | **ab** | | | 2,66 | | | **b** | | 2,58 | | | | **b** | | | 1,68 | | | **ab** | | | | ******* | | |  |  |  |  |  |  |
| Hotrienol | T16 | 1609/1610 | RI/MS | 0,41 | | **b** | | 0,00 | | **a** | | 0,00 | | **a** | 0,00 | | **a** | | 0,00 | | **a** | | 0,00 | | **a** | | | 0,00 | | | **a** | | | 0,25 | | | **b** | | 0,88 | | | | **c** | | | 0,00 | | | **a** | | | | ******* | | |  |  |  |  |  |  |
| β-Cyclocitral | T17 | 1622/1623 | RI/MS | 1,05 | | **abc** | | 1,29 | | **abc** | | 3,55 | | **abc** | 1,48 | | **abc** | | 0,80 | | **ab** | | 2,77 | | **abc** | | | 4,15 | | | **bc** | | | 5,05 | | | **c** | | 2,89 | | | | **bc** | | | 0,00 | | | **a** | | | | ******* | | |  |  |  |  |  |  |
| Menthol | T18 | 1640/1641 | RI/MS | 0,00 | | **a** | | 0,00 | | **a** | | 0,56 | | **ab** | 3,22 | | **ab** | | 1,23 | | **ab** | | 0,65 | | **ab** | | | 0,00 | | | **a** | | | 3,03 | | | **ab** | | 1,92 | | | | **ab** | | | 3,47 | | | **b** | | | | ******* | | |  |  |  |  |  |  |
| α-Terpineol | T19 | 1703/1702 | RI/MS | 58,70 | | **ab** | | 10,19 | | **a** | | 21,01 | | **a** | 16,32 | | **a** | | 8,59 | | **a** | | 16,00 | | **a** | | | 44,63 | | | **a** | | | 112,84 | | | **ab** | | 162,01 | | | | **b** | | | 30,19 | | | **a** | | | | ******* | | |  |  |  |  |  |  |
| Cital | T20 | 1739/1733 | RI/MS | 0,30 | | **a** | | 0,18 | | **a** | | 0,30 | | **a** | 0,17 | | **a** | | 0,19 | | **a** | | 0,17 | | **a** | | | 0,26 | | | **a** | | | 0,81 | | | **ab** | | 1,31 | | | | **b** | | | 0,58 | | | **ab** | | | | ******* | | |  |  |  |  |  |  |
| Nerol | T21 | 1801/1800 | RI/MS | 1,86 | | **a** | | 0,29 | | **a** | | 0,58 | | **a** | 0,56 | | **a** | | 0,26 | | **a** | | 0,42 | | **a** | | | 1,11 | | | **a** | | | 3,25 | | | **ab** | | 5,62 | | | | **b** | | | 1,01 | | | **a** | | | | ******* | | |  |  |  |  |  |  |
| Dihydro β-ionone | T22 | 1835/1842 | RI/MS | 0,00 | | **a** | | 1,30 | | **a** | | 14,59 | | **c** | 9,72 | | **bc** | | 2,20 | | **a** | | 3,87 | | **ab** | | | 6,09 | | | **ab** | | | 1,96 | | | **a** | | 0,00 | | | | **a** | | | 0,00 | | | **a** | | | | ******* | | |  |  |  |  |  |  |
| Geraniol | T23 | 1850/1851 | RI/MS | 5,27 | | **a** | | 1,44 | | **a** | | 2,04 | | **a** | 1,81 | | **a** | | 0,98 | | **a** | | 1,83 | | **a** | | | 4,34 | | | **a** | | | 10,56 | | | **ab** | | 17,87 | | | | **b** | | | 3,19 | | | **a** | | | | ******* | | |  |  |  |  |  |  |
| Geranyl acetone | T24 | 1860/1862 | RI/MS | 0,63 | | **a** | | 0,57 | | **a** | | 1,04 | | **a** | 0,86 | | **a** | | 0,95 | | **a** | | 0,81 | | **a** | | | 1,10 | | | **a** | | | 2,37 | | | **a** | | 3,67 | | | | **b** | | | 1,68 | | | **a** | | | | ******* | | |  |  |  |  |  |  |
| β-Ionone | T25 | 1952/1953 | RI/MS | 0,59 | | **a** | | 1,17 | | **ab** | | 3,33 | | **bc** | 2,06 | | **abc** | | 0,69 | | **a** | | 1,77 | | **abc** | | | 2,96 | | | **abc** | | | 3,84 | | | **c** | | 3,49 | | | | **bc** | | | 1,77 | | | **abc** | | | | ******* | | |  |  |  |  |  |  |
| 2-Methyl butanal | A1 | 899/899 | RI/MS | 0,64 | | **ab** | | 1,41 | | **ab** | | 1,35 | | **ab** | 1,29 | | **ab** | | 1,00 | | **ab** | | 1,55 | | **ab** | | | 1,70 | | | **b** | | | 0,00 | | | **a** | | 0,70 | | | | **ab** | | | 1,80 | | | **b** | | | | ***** | | |  |  |  |  |  |  |
| Hexanal | A2 | 1088/1086 | RI/MS | 22,08 | |  | | 11,05 | |  | | 28,19 | |  | 17,57 | |  | | 24,81 | |  | | 52,40 | |  | | | 69,15 | | |  | | | 17,24 | | |  | | 45,66 | | | |  | | | 23,67 | | |  | | | | **ns** | | |  |  |  |  |  |  |
| 2-Hexenal | A3 | 1242/1248 | RI/MS | 102,16 | |  | | 26,49 | |  | | 63,08 | |  | 45,90 | |  | | 39,00 | |  | | 74,82 | |  | | | 136,60 | | |  | | | 69,53 | | |  | | 75,95 | | | |  | | | 70,59 | | |  | | | | **ns** | | |  |  |  |  |  |  |
| Benzaldehyde | A4 | 1530/1532 | RI/MS | 3,06 | |  | | 45,07 | |  | | 9,81 | |  | 2,28 | |  | | 3,14 | |  | | 2,32 | |  | | | 2,36 | | |  | | | 5,37 | | |  | | 4,06 | | | |  | | | 2,08 | | |  | | | | **ns** | | |  |  |  |  |  |  |
| Benzeneacetaldehyde | A5 | 1645/1646 | RI/MS | 2,18 | | **ab** | | 0,00 | | **a** | | 0,75 | | **a** | 2,72 | | **ab** | | 0,00 | | **a** | | 0,99 | | **ab** | | | 2,21 | | | **ab** | | | 3,80 | | | **ab** | | 3,23 | | | | **ab** | | | 4,68 | | | **b** | | | | ****** | | |  |  |  |  |  |  |
| 2-Pentanone-4-methyl | K1 | 1011/1012 | RI/MS | 0,25 | | **b** | | 0,00 | | **a** | | 0,00 | | **a** | 0,00 | | **a** | | 0,00 | | **a** | | 0,00 | | **a** | | | 0,00 | | | **a** | | | 0,00 | | | **a** | | 0,00 | | | | **a** | | | 0,00 | | | **a** | | | | ******* | | |  |  |  |  |  |  |
| 3-Octanone | K2 | 1275/1277 | RI/MS | 0,41 | | **a** | | 0,91 | | **a** | | 0,82 | | **a** | 1,05 | | **a** | | 0,00 | | **a** | | 1,47 | | **a** | | | 1,34 | | | **a** | | | 1,55 | | | **a** | | 4,59 | | | | **b** | | | 5,77 | | | **b** | | | | ******* | | |  |  |  |  |  |  |
| 6-Methyl-5-hepten-2-one | K3 | 1350/1348 | RI/MS | 9,16 | | **ab** | | 17,26 | | **b** | | 5,11 | | **a** | 3,95 | | **a** | | 1,72 | | **a** | | 4,30 | | | **a** | | | 7,67 | | | | **ab** | | 7,63 | | **ab** | | | 12,76 | | | | **ab** | | | 11,82 | | | | **ab** | | | ****** | | |  |  |  |  |  |
| γ-Caprolactone | L1 | 1707/1706 | | | RI/MS | | 0,93 | | **a** | | 0,00 | | **a** | 2,68 | | **ab** | | 1,84 | | **a** | | 1,46 | | **a** | | | 4,99 | | | **ab** | | 0,69 | | | | **a** | | 6,53 | | | **b** | | | | 0,72 | | | **a** | | | | 1,34 | | | **a** | | | | ******* | | |
| γ-Octalactone | L2 | 1932/1937 | | | RI/MS | | 0,74 | | **a** | | 0,33 | | **a** | 4,62 | | **ab** | | 2,31 | | **a** | | 1,88 | | **a** | | | 7,25 | | | **ab** | | 1,40 | | | | **a** | | 12,42 | | | **b** | | | | 0,98 | | | **a** | | | | 1,97 | | | **a** | | | | ******* | | |
| δ-Octalactone | L3 | 1976/1975 | | | RI/MS | | 0,00 | | **a** | | 0,00 | | **a** | 0,58 | | **ab** | | 0,00 | | **a** | | 0,22 | | **ab** | | | 0,81 | | | **b** | | 0,00 | | | | **a** | | 0,87 | | | **b** | | | | 0,00 | | | **a** | | | | 0,00 | | | **a** | | | | ******* | | |
| γ-Heptalactone/Nonalactone | L4 |  | | | RI/MS | | 0,00 | | **a** | | 0,00 | | **a** | 0,50 | | **ab** | | 0,49 | | **ab** | | 0,53 | | **ab** | | | 0,63 | | | **ab** | | 0,34 | | | | **ab** | | 1,09 | | | **b** | | | | 0,43 | | | **ab** | | | | 0,22 | | | **ab** | | | | ******* | | |
| γ-Decalactone | L5 |  | | | RI/MS | | 6,46 | | **a** | | 0,86 | | **a** | 53,66 | | **ab** | | 68,64 | | **ab** | | 49,68 | | **ab** | | | 115,26 | | | **b** | | 3,77 | | | | **a** | | 87,91 | | | **ab** | | | | 2,70 | | | **a** | | | | 5,66 | | | **a** | | | | ******* | | |
| δ-Octalactone/Nonalactone/Decalactone | L6 |  | | | RI/MS | | 0,48 | | **a** | | 0,00 | | **a** | 6,17 | | **a** | | 3,72 | | **a** | | 5,73 | | **a** | | | 16,83 | | | **b** | | 0,00 | | | | **a** | | 6,49 | | | **a** | | | | 0,36 | | | **a** | | | | 0,00 | | | **a** | | | | ******* | | |
| γ-Dodecalactone | L7 |  | | | RI/MS | | 0,00 | | **a** | | 0,00 | | **a** | 2,34 | | **ab** | | 0,64 | | **a** | | 2,27 | | **ab** | | | 5,73 | | | **b** | | 0,00 | | | | **a** | | 2,08 | | | **ab** | | | | 0,00 | | | **a** | | | | 0,21 | | | **a** | | | | ******* | | |
| Hexane | H1 | 612/600 | | | RI/MS | | 0,48 | | **a** | | 4,66 | | **b** | 0,23 | | **a** | | 0,42 | | **a** | | 0,82 | | **a** | | | 0,45 | | | **a** | | 0,34 | | | | **a** | | 0,00 | | | **a** | | | | 0,63 | | | **a** | | | | 1,05 | | | **a** | | | | ******* | | |
| Dodecane | H2 | 1208/1200 | | | RI/MS | | 0,17 | | **b** | | 0,00 | | **a** | 0,00 | | **a** | | 0,00 | | **a** | | 0,00 | | **a** | | | 0,00 | | | **a** | | 0,00 | | | | **a** | | 0,00 | | | **a** | | | | 0,00 | | | **a** | | | | 0,00 | | | **a** | | | | ******* | | |
| 4-Methyl-1,3-pentadiene | O1 | 755/746 | | | RI/MS | | 0,16 | | **b** | | 0,27 | | **c** | 0,00 | | **a** | | 0,00 | | **a** | | 0,00 | | **a** | | | 0,00 | | | **a** | | 0,00 | | | | **a** | | 0,00 | | | **a** | | | | 0,00 | | | **a** | | | | 0,00 | | | **a** | | | | ******* | | |
| Naphtalene 1,2-dihydro-1,1,6-trimethyl | O2 | 1748/1747 | | | RI/MS | | 0,00 | | **a** | | 0,09 | | **a** | 0,00 | | **a** | | 0,00 | | **a** | | 0,12 | | **a** | | | 0,12 | | | **a** | | 0,00 | | | | **a** | | 0,30 | | | **a** | | | | 0,00 | | | **a** | | | | 0,00 | | | **a** | | | | ******* | | |
| Formamide N,N-dibutyl | O3 | 1779/1773 | | | RI/MS | | 0,00 | |  | | 1,80 | |  | 0,76 | |  | | 0,24 | |  | | 0,71 | |  | | | 0,00 | | |  | | 0,44 | | | |  | | 1,04 | | |  | | | | 2,12 | | |  | | | | 0,65 | | |  | | | | **ns** | | |
| Benzothiazole | O4 | 1962/1968 | | | RI/MS | | 0,65 | | **a** | | 0,68 | | **a** | 0,45 | | **a** | | 0,31 | | **a** | | 0,55 | | **a** | | | 0,51 | | | **a** | | 0,59 | | | | **a** | | 2,59 | | | **b** | | | | 0,87 | | | **a** | | | | 0,00 | | | **a** | | | | ******* | | |
